# Quercetin promotes bone marrow mesenchymal stem cell proliferation and osteogenic differentiation through the H19/miR-625-5p axis to activate the Wnt/β-catenin pathway

**DOI:** 10.1186/s12906-021-03418-8

**Published:** 2021-09-30

**Authors:** Wei Bian, Shunqiang Xiao, Lei Yang, Jun Chen, Shifang Deng

**Affiliations:** 1 Department of Traditional Chinese Medicine, Shenzhen People’s Hospital (The Second Clinical Medical College, Jinan University, The First Affiliated Hospital, Southern University of Science and Technology), No. 1017, Dongmen North Road, Luohu District, Shenzhen, 518020 China; 2 Department of Geriatrics in Luohu Hospital of Traditional Chinese Medicine/Shenzhen Hospital of Shanghai University of traditional Chinese Medicine, Shenzhen, 518000 China

**Keywords:** lncRNA, microRNAs, Osteogenic differentiation, Quercetin

## Abstract

**Background:**

Quercetin and H19 can promote osteogenic differentiation of bone marrow mesenchymal stem cells (BMSCs). However, whether quercetin regulates H19 expression to promote osteogenic differentiation of BMSCs is unclear.

**Methods:**

BMSC proliferation, matrix mineralization, and alkaline phosphatase (ALP) activity were assessed using the Cell Counting Kit-8, ALP assay kit, and alizarin red staining kit, respectively. Expression of H19, miR-625-5p, BMP-2, osteocalcin, and RUNX2 were measured by qRT-PCR; β-catenin protein level was measured by western blotting.

**Results:**

Quercetin promoted BMSC proliferation, enhanced ALP activity, and upregulated the expression of BMP-2, osteocalcin, and RUNX2 mRNAs, suggesting that it promoted osteogenic differentiation of BMSCs. Moreover, quercetin increased H19 expression, while the effect of quercetin on BMSCs was reversed by silencing H19 expression. Additionally, miR-625-5p, interacted with H19, was downregulated during quercetin-induced BMSC osteogenic differentiation, which negatively correlated with H19 expression. Silencing miR-625-5p expression promoted BMSC proliferation and osteogenic differentiation, whereas miR-625-5p overexpression weakened the effect of quercetin on BMSCs. Finally, quercetin treatment or downregulation of miR-625-5p expression increased β-catenin protein level in BMSCs. Upregulation or downregulation of miR-625-5p or H19 expression, respectively, inhibited β-catenin protein level in quercetin treated-BMSCs.

**Conclusion:**

H19 promotes, while miR-625-5p inhibits BMSC osteogenic differentiation. Quercetin activates the Wnt/β-catenin pathway and promotes BMSC osteogenic differentiation via the H19/miR-625-5p axis.

**Supplementary Information:**

The online version contains supplementary material available at 10.1186/s12906-021-03418-8.

## Background

Human bone marrow mesenchymal stem cells (BMSCs) can undergo self-renewal and osteogenic differentiation, and play a vital function for bone formation and remodeling [1]. Some pathophysiological conditions, such as aging, osteoporosis, and some bone defects can inhibit the osteogenic ability of BMSCs [2–4]. Therefore, drugs that can regulate BMSC osteogenic differentiation need to be developed for the management and treatment of these orthopedic diseases.

Quercetin, a naturally available flavonoid and a well-known phytoestrogen [5], exerts antioxidant and anti-inflammatory properties. In vitro, quercetin promotes osteogenic differentiation of BMSCs via extracellular-signal-regulated protein kinases, adenosine 5'-monophosphate (AMP)-activated protein kinase/sirtuin 1, and estrogen receptor-mediated pathways [6–8]. In vivo, in rat models of postmenopausal osteoporosis, quercetin heightens BMSC osteogenic differentiation to increase the bone mineral density [9]. However, currently available scientific data has not yet elucidated the functional mechanism of quercetin. Therefore, further understanding of the mechanisms underlying quercetin effects will have immense clinical implications.

Long non-coding RNAs (lncRNAs) can regulate protein levels via epigenetic modulations. LncRNAs affect several biological processes, including bone metabolism and osteogenic differentiation of BMSCs [10, 11]. According to lncRNA microarray results reported by Wang et al and Zhang et al, lncRNAs upregulate H19 levels during osteogenic differentiation [12, 13], which in turn, promote osteogenic differentiation of BMSCs [14, 15]. Furthermore, quercetin promotes the apoptosis of prostate cancer cells, colorectal cancer cells, and fibroblast-like synoviocytes by regulating lncRNA expression [16–18]. However, it remains unclear whether quercetin can promote BMSC osteogenic differentiation by regulating H19 expression.

Therefore, in this study, the effect of quercetin and H19 expression on the proliferation and osteogenic differentiation of BMSCs was identified. Further, the mechanism was elucidated by which quercetin affects BMSC osteogenic differentiation.

## Material and methods

### BMSCs culture, quercetin treatment, and siRNA transfection

Human BMSCs (catalog number. ZQ0308), complete medium (No. 7501), and osteogenic differentiation medium (No. 7531) were purchased from Zhong-Qiao-Xin-Zhou (Shanghai, China). Quercetin (Aladdin, No: Q111273, purity ≥98.5%, Shanghai, China) was dissolved in DMSO. BMSCs were cultured in complete medium supplemented with 50 μg/mL ascorbic acid and 10 mM β-glycerophosphate (CM medium) and then treated with different concentrations of quercetin (1, 5, and 10 μmol/L). BMSCs in the negative control group were treated with DMSO; BMSCs in the positive control group (positive group) were treated with osteogenic differentiation medium. H19 siRNA (si-H19), negative control siRNA (si-NC), miR-625-5p mimic, miR-625-5p inhibitor, NC mimic, and NC inhibitor were synthesized by Genepharma (Shanghai, China) and transfected into BMSCs.

### Proliferation assay

BMSCs (1×10^4^ cells/100 μL/well) were cultured for 24 hours. Once BMSCs adhered, BMSCs were treated with DMSO or quercetin (1, 5, and 10 μM) in osteogenic differentiation medium. After 1, 2, and 7 days of treatment, 10 μl CCK-8 solution (Dojindo, Japan) was added and incubated for 4 hours. Finally, optical density at 450 nm (OD_450_) was measured using a Multiskan Mk3 microplate reader (Thermo Fisher).

### Alkaline phosphatase assay

An alkaline phosphatase (ALP) assay kit (Beyotime, Shanghai, China) was used to assess the ALP activity. Briefly, BMSCs were lysed and diluted with cell lysate. Standard substance in this kit was added in volumes of 4 μL (20 nmol/L ALP), 8 μL (40 nmol/L), 16 μL (80 nmol/L ALP), 24 μL (120 nmol/L ALP), 32 μL (160 nmol/L ALP), and 40 μL (200 nmol/L ALP), and 50 μL of the lysates was added to the 96-well plate. After mixing, the 96-well plate was incubated at 37 °C. The ALP activity was calculated as the p-nitrophenol produced per min per milligram of protein (unit: nmol/min/mg). The blank acted as negative control.

### Matrix mineralization

BMSCs were cultured in CM medium or osteogenic differentiation medium at 37 ^o^C in a 5% CO_2_ incubator. Every 3 days, fresh medium (preheated to 37 ^o^C) was used to replace the old CM medium. After 21 days of quercetin treatment, the medium was removed, BMSCs were stained with Alizarin Red solution (Aladdin, Shanghai, China). Finally, the BMSCs were washed and images were acquired under a microscope (Beijing Cnmicro Instrument Co., Ltd., Beijing, China).

### Quantitative real-time polymerase chain reaction (qRT-PCR)

qRT-PCR was performed to analyze the expression of H19, miR-625-5p, bone morphogenetic protein 2 (BMP-2), osteocalcin, and runt-related transcription factor 2 (RUNX2). Briefly, total extractive RNA was reverse-transcribed using EasyScript First-Strand cDNA Synthesis SuperMix (TransGen Biotech, Beijing, China) and the miRNA First Strand cDNA Synthesis Kit (Sangon Biotech Co., Ltd. Shanghai, China). qRT-PCR was performed using the SYBR Green qPCR SuperMix (Invitrogen) on the ABI PRISM® 7500 Sequence Detection System (Foster City, CA, USA). Relative expression levels of lncRNA, miRNA, and miRNA expression were determined using the 2^−ΔΔct^ method [19], according to internal control which used 18s RNA and U6 levels. The information related to the primers used is listed in Table 1.

**Table 1:** Sequences of primers used in the study

Gene	Sequence (5′ -3′)	Size
miR-625-5p-Forward	ACTCCAGCTGGGAGGGGGAAAGTTCTATAG	71 bp
miR-625-5p-Reverse	CTCAACTGGTGTCGTGGA	
U6- Forward	CTCGCTTCGGCAGCACA	96 bp
U6- Reverse	AACGCTTCACGAATTTGCGT	
BMP2-Forward	ACGCCTTAAGTCCAGCTGTA	160 bp
BMP2-Reverse	GGCATGATTAGTGGAGTTCA	
Runx2- Forward	TCTAAATCGCCAGGCTTCAT	250 bp
Runx2-Reverse	GAGGACCTACTCCCAAAGGA	
Osteocalcin- Forward	CTCACACTCCTCGCCCTATT	139 bp
Osteocalcin-Reverse	TGGGTCTCTTCACTACCTCG	
H19-Forward	GCGGGTCTGTTTCTTTACTTC	171 bp
H19-Reverse	GTGGTTGTAAAGTGCAGCAT	
18s-Forward	CCTGGATACCGCAGCTAGGA	112 bp
18s-Reverse	GCGGCGCAATACGAATGCCCC	

### Bioinformatic database assay and luciferase reporter assay

LncBase Experimental v.2 and Starbase 3.0 were used to find the potential miRNAs binding to H19 [20, 21]. Abnormally expressed miRNAs during BMSC osteogenic differentiation were analyzed using GEO2R in GSE148049 of the Gene Expression Omnibus (https://www.ncbi.nlm.nih.gov/geo/). The potential miRNAs at the intersection of three sets of results were selected.

293T cells (5×10^4^ cells/well) were plated and cultured for 24 h. The binding of H19 to miRNAs was analyzed using the luciferase reporter assay. Briefly, the wild type H19 (H19-WT) and mutant H19 (H19-Mut, with mutated binding sites) sequences were cloned onto the luciferase reporter vector psi-CHECK2 and then transfected into 293T cells. Forty-eight hours later, the luciferase activity of renilla or firefly luciferase activity was evaluated by the dual luciferase reporter assay system (Promega). The renilla/firefly luciferase activity rate was lower in the co-transfected H19 and miR-625-5p mimic groups than that in co-transfected NC mimic and WT-H19 groups, suggesting that H19 can bind to miR-625-5p. The renilla/firefly luciferase rate did not change significantly between the co-transfected H19 and miR-625-5p mimic groups and the co-transfected NC mimic and WT-H19 groups, suggesting that H19 cannot bind to miR-625-5p.

### Western blot analysis

The extractive total proteins were separated by performing SDS-PAGE using 10% resolving gels, then transferred onto polyvinylidene fluoride membranes and incubated with rabbit monoclonal antibody specific to β-catenin (dilution, 1:500, ab68183, Abcam, San Diego, CA, USA) and rabbit monoclonal antibody specific to GAPDH (dilution, 1:10,000, ab128915, Abcam) for 40 min. The membranes were washed three times and incubated with Goat Anti-Rabbit IgG H&L (HRP) (dilution, 1:10,000, ab205718, Abcam). Finally, protein bands were visualized using an enhanced chemiluminescent reagent (PerkinElmer Life Sciences, MA, USA).

### Statistical analysis

Data, conform to normal distribution, are presented as the mean ± standard deviation. One-way analysis of variance was performed using SPSS 19.0 statistical software (IBM, Inc.) to analyze the statistical difference between more than three groups, followed by Tukey’s post-hoc test. Statistical significance was set at *P* < 0.05.

## Results

### Quercetin enhances BMSC osteogenic differentiation

**Fig. 1:**
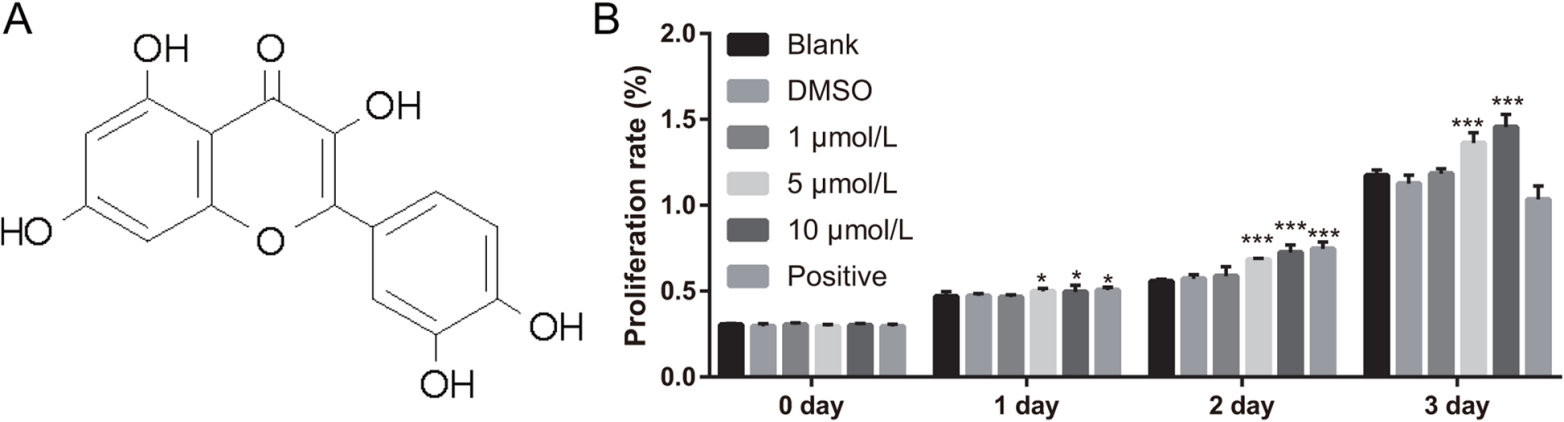
Quercetin enhanced BMSC proliferation. **A** Structural formula of quercetin; **B** BMSC proliferation was enhanced on days 1, 2, and 7 after they were treated with quercetin and osteogenic differentiation medium (positive group). *P < 0.05, **P < 0.01, and ***P < 0.001; vs Blank group

**Fig. 2:**
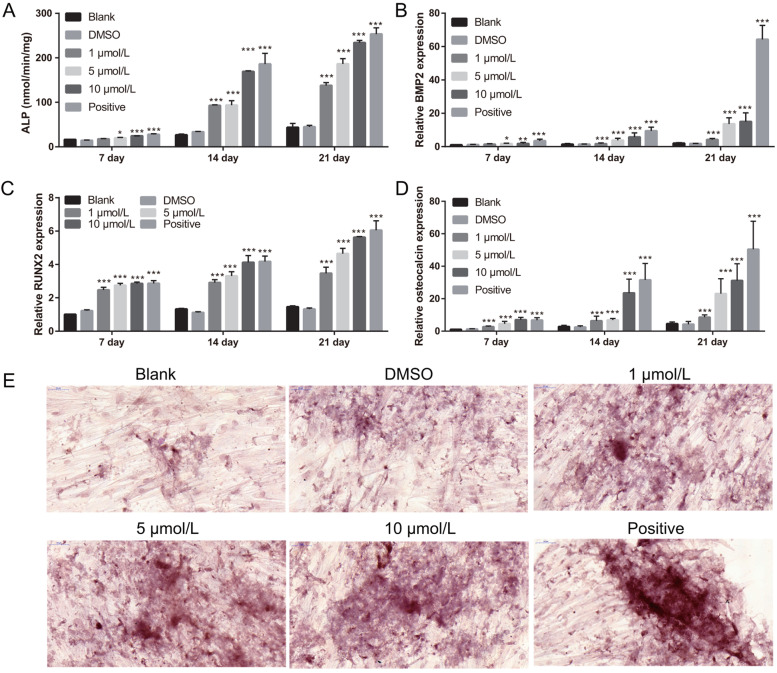
Quercetin enhanced ALP activity; BMP-2, RUNX2, and osteocalcin mRNA expression; and calcium nodule formation. **A** ALP activity was enhanced at days 1, 2, and 7 after treatment with quercetin and osteogenic differentiation medium (positive group). **B-D** BMP-2, RUNX2, and osteocalcin mRNA expression were enhanced on days 1, 2, and 7 after treatment with quercetin and osteogenic differentiation medium (positive group). **E** Calcium nodule formation was enhanced on days 1, 2, and 7 after treatment with quercetin and osteogenic differentiation medium (positive group) (400×). *P < 0.05, **P < 0.01, and ***P < 0.001; vs Blank group

The molecular structural formula of quercetin is shown in Fig. 1A. Cell proliferation increased significantly in quercetin treatment groups (both 5 and 10 μM) compared with that in the blank group from day 1 to day 7, whereas cell proliferation in the positive group increased significantly on day 1 and 2 but decreased on day 7 (Fig. 1B). ALP activity and the transcription of BMP-2, osteocalcin, and RUNX2 was significantly enhanced in the quercetin treatment and positive groups compared with those in the blank group (Fig. 2A–D). Additionally, calcium nodules were observed in all groups after treatment for 21 days (Fig. 2E). Compared with that in the blank group, the number and area of calcium nodules notably increased in the quercetin treatment and positive groups (Fig. 2E). These results suggest that quercetin treatment significantly increased cell proliferation and osteogenic differentiation.

### Quercetin promotes H19 expression

**Fig. 3:**
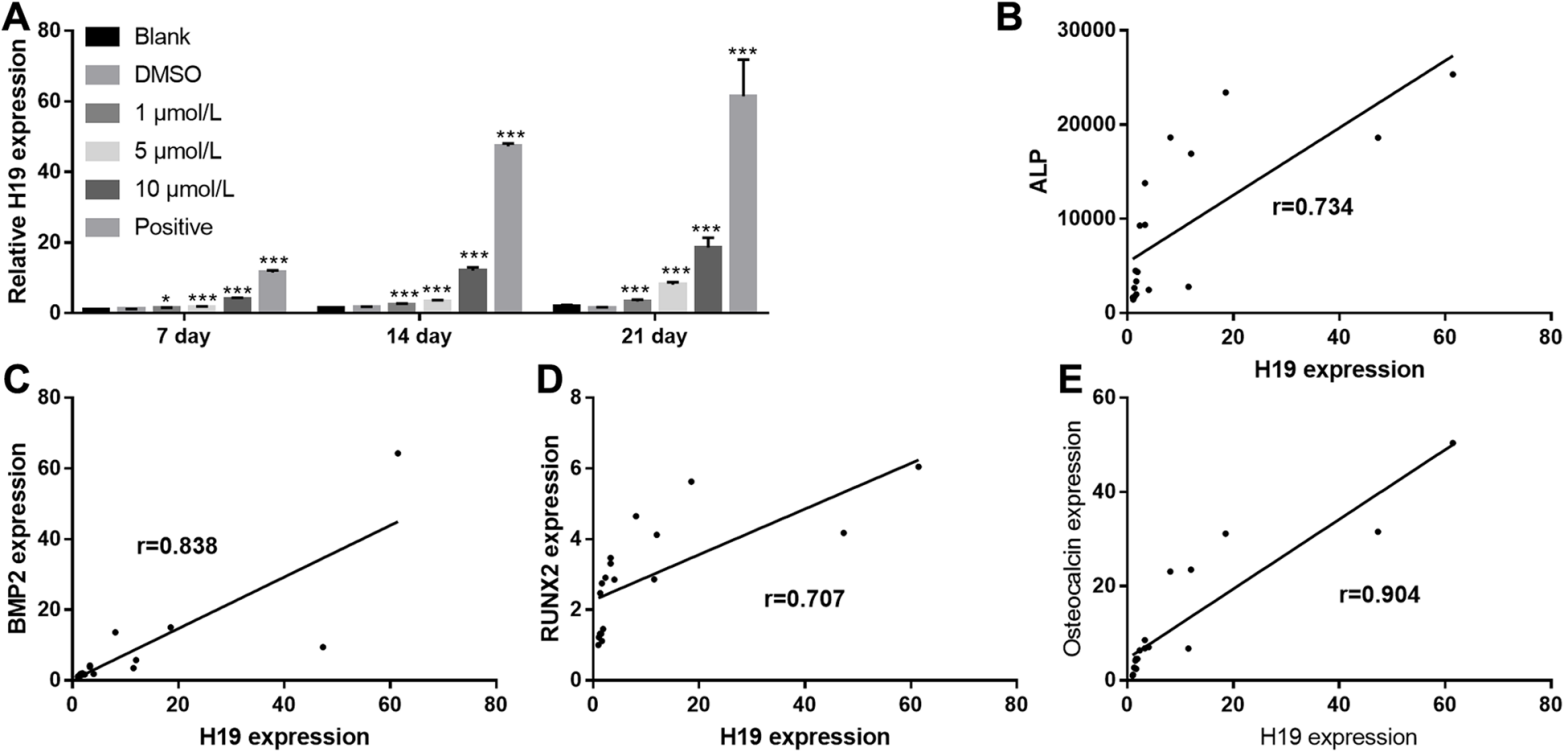
H19 expression was enhanced during quercetin-induced BMSC osteogenic differentiation; this enhanced expression had a positive relationship with alkaline phosphatase activity, and BMP-2, RUNX2, and osteocalcin mRNA expression. **A** H19 expression was enhanced on days 1, 2, and 7 after treatment with quercetin and osteogenic differentiation medium (positive group). *P < 0.05, **P < 0.01, and ***P < 0.001; vs Blank group. **B-E** H19 expression had a positive relationship with alkaline phosphatase activity, **B** and BMP-2 **C**, RUNX2 **D**, and osteocalcin **E** mRNA expression

The H19 levels was measured by performing qRT-PCR after treating cells with quercetin and osteogenic differentiation medium (positive group). H19 expression increased significantly in the quercetin treatment and positive groups compared with that observed in the blank group (Fig. 3A). Furthermore, H19 expression had a positive relationship with ALP activity, and the mRNA expression of BMP-2, RUNX2, and osteocalcin (Fig. 3B–E).

### Silencing H19 expression reverses the effect of quercetin on BMSCs

**Fig. 4:**
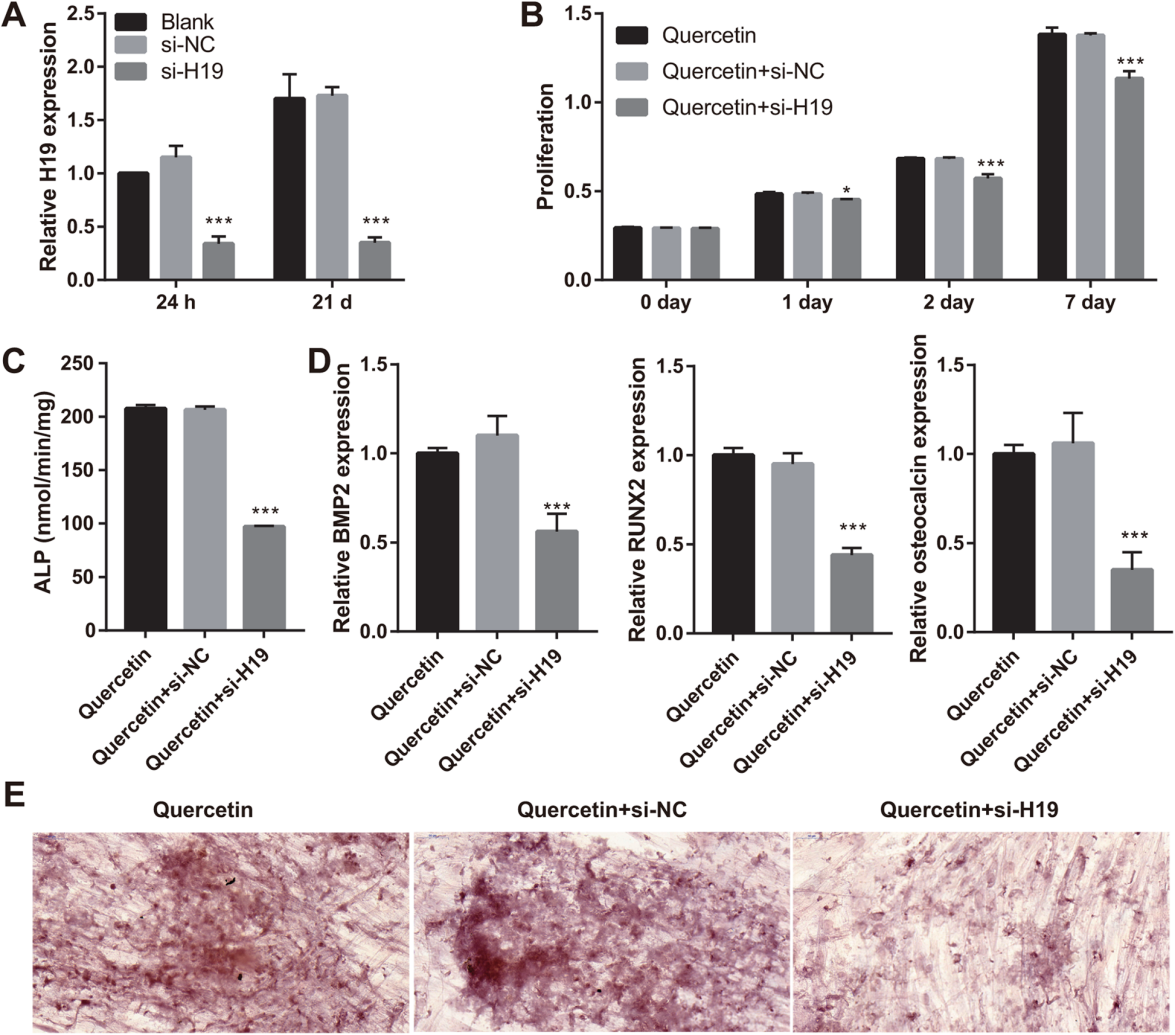
Silencing H19 expression inhibited BMSC proliferation; alkaline phosphatase activity; BMP-2, RUNX2, and osteocalcin mRNA expression; and calcium nodule formation in quercetin-treated BMSCs. **A** H19 expression was inhibited after si-H19 transfection at 24 h and 21 days. **B** Transfected BMSC proliferation decreased on days 1, 2, and 7 after quercetin treatment. **C** Alkaline phosphatase (ALP) activity reduced after si-H19 transfection and quercetin treatment for 21 days; **D** BMP-2, RUNX2, and osteocalcin mRNA expression decreased after si-H19 transfection and quercetin treatment for 21 days. **E** Calcium nodule formation was reduced after si-H19 transfection and quercetin treatment for 21 days (400×). *P < 0.05 and ***P < 0.001 vs si-NC group

To study the effect of H19, BMSCs were transfected with si-H19, followed by treatment with 10 μM quercetin; si-NC was used as control. H19 expression was successfully silenced by si-H19 (Fig. 4A). Following H19 silencing, the proliferation of BMSCs was inhibited on days 1, 2, and 7 (Fig. 4B). Moreover, on day 21 after H19 silencing, ALP activity and mRNA levels of BMP-2, RUNX2, and osteocalcin were significantly reduced (Fig. 4C–D). The number and area of calcium nodules were notably decreased following H19 silencing on day 21 (Fig. 4E).

### H19 interacts with miR-625-5p

**Fig. 5:**
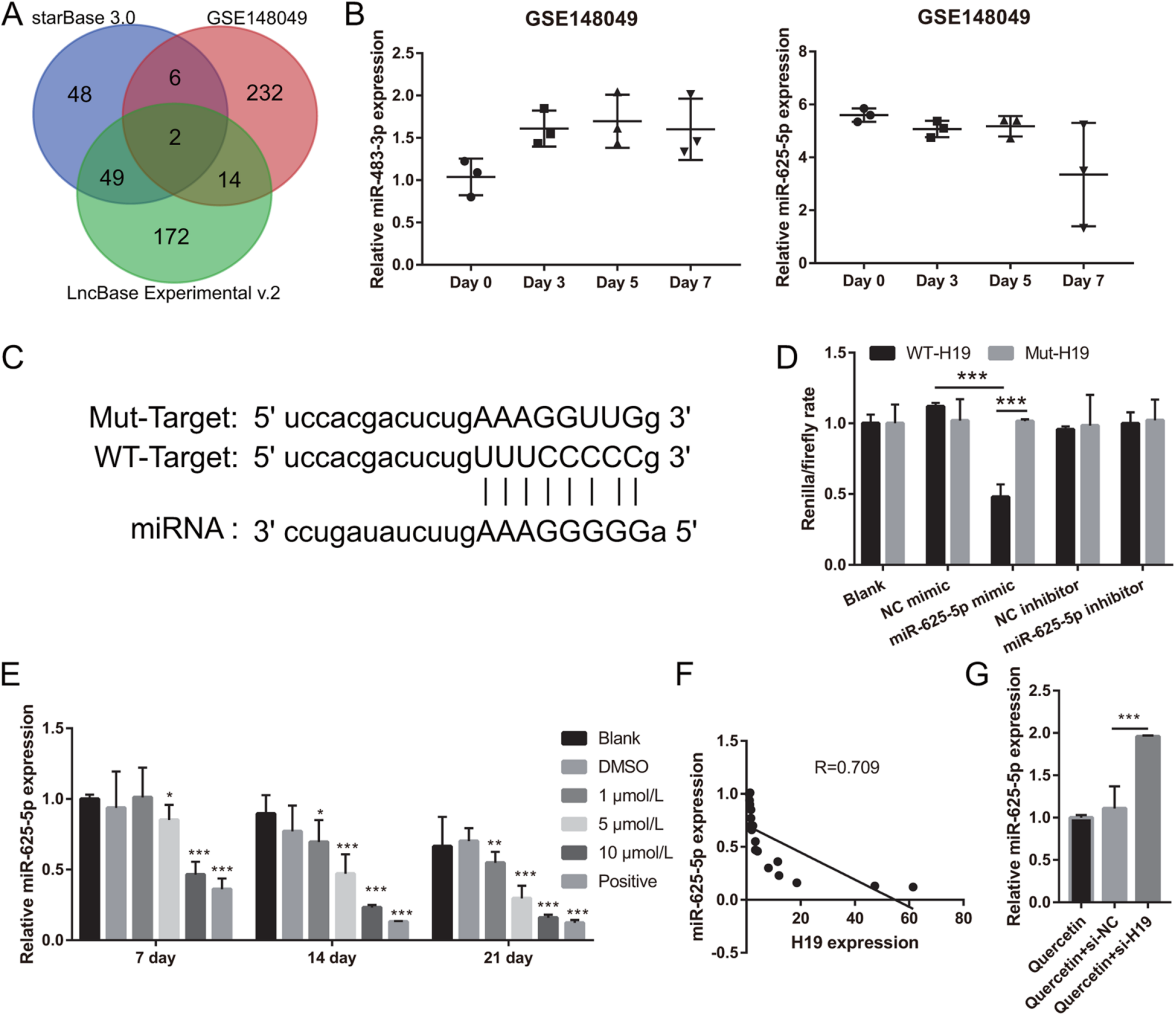
H19 interacts with miR-625-5p and inhibits miR-625-5p expression. **A** miR-483-3p and miR-625-5p were selected from the intersection of the GSE148049 dataset, Starbase 3.0, and LncBase Experimental v.2 analysis results. Differentially expressed miRNAs during BMSC osteogenic differentiation were identified from by the GSE148049 dataset. Potential miRNAs binding to *H19* were determined according to Starbase 3.0 and LncBase Experimental v.2 analysis. **B** miR-483-3p and miR-625-5p expression were analyzed in the GSE148049 dataset. ***P < 0.001 vs Day 0. **C** The binding sites between H19 and miR-625-5p were analyzed by Starbase 3.0. **D** The binding of H19 to miR-625-5p was measured by the luciferase reporter assay. ***P < 0.001. **E** miR-625-5p expression reduced during quercetin-induced BMSC osteogenic differentiation. **P < 0.01 and ***P < 0.001, vs Blank group. **F** miR-625-5p expression negatively correlated with H19 expression. **G** miR-625-5p expression increased at 21 days after transfection. ***P < 0.001

Firstly, we identified 254 miRNAs in the GSE148049 dataset that were differentially expressed during BMSC osteogenic differentiation. Additionally, 105 and 237 potential miRNAs binding to *H19* were identified. miR-625-5p and miR-483-3p were selected through the intersection of the three sets of results (Fig. 5A). Although miR-625-5p and miR-483-3p expression did not change significantly between day 0 and day 7 during BMSC osteogenic differentiation (*P*=0.064>0.05 and *P*=0.097>0.05, respectively), miR-625-5p expression was higher while that of miR-483-3p was lower on day 7 than on day 0 (Fig. 5B). This result suggested that miR-483-3p promotes BMSC osteogenic differentiation, whereas miR-625-5p inhibits BMSC osteogenic differentiation. Since H19 plays a role in promoting osteogenic differentiation, we aimed to identify miRNAs that inhibit osteogenic differentiation to elucidate the function of H19. Therefore, miR-625-5p was selected for further evaluation. The binding site between H19 and miR-625-5p is shown in Fig. 5C. Renilla/firefly luciferase activity rate was lower in the miR-625-5p mimic+WT-H19 group than in the NC mimic+WT-H19 group, and it comparable between the NC+Mut-H19 group and miR-625-5p mimic+Mut-H19 group, suggesting that H19 can bind to miR-625-5p (Fig. 5D). Finally, we observed that miR-625-5p expression gradually decreased during quercetin-induced BMSC osteogenic differentiation and negatively correlated with H19 expression (Fig. 5E and F). During quercetin-induced BMSC osteogenic differentiation, downregulation of H19 expression promoted miR-625-5p expression (Fig. 5G).

### miR-625-5p overexpression reverses the effect of quercetin on BMSCs

**Fig. 6:**
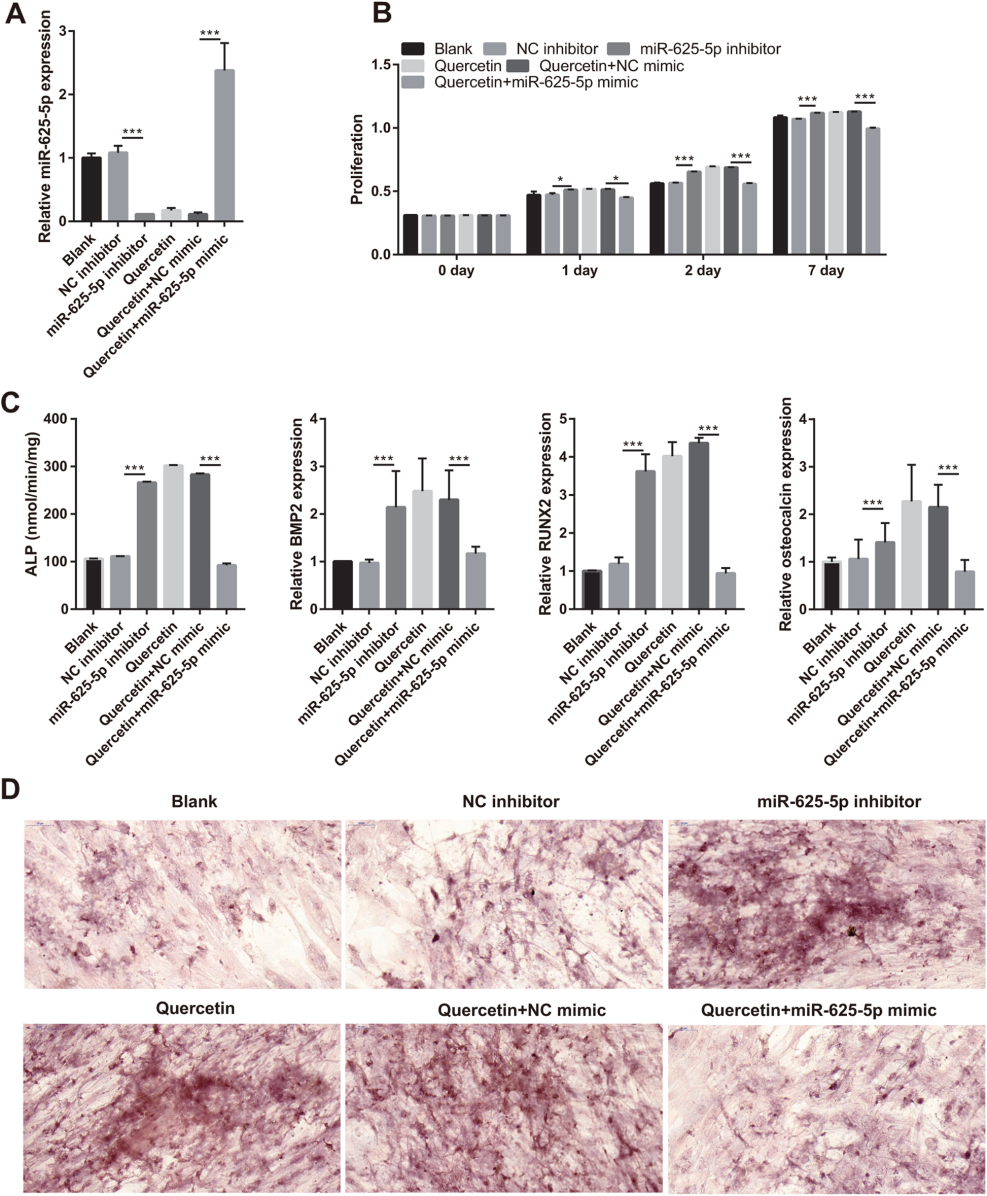
miR-625-5p overexpression reversed quercetin effects on BMSCs. **A** miR-625-5p expression was measured by qRT-PCR at 24 h after transfection. **B** Proliferation of transfected BMSCs was measured using the Cell Counting Kit-8 on days 1, 2, and 7 following quercetin treatment. **C** ALP activity was measured using the Alkaline Phosphatase Assay Kit, and BMP-2, RUNX2, and osteocalcin mRNA expressions were measured by qRT-PCR after transfection and quercetin treatment for 21 days. **D** Calcium nodule formation was detected using Alizarin Red staining (400×) after transfection and quercetin treatment for 21 days. *P < 0.05 and ***P < 0.001

Next, miR-625-5p inhibitor was transfected into BMSCs to decrease the miR-625-5p level, while the transfection of NC inhibitor was used as control. miR-625-5p level was successfully decreased by miR-625-5p inhibitor (Fig. 6A), which promoted BMSC proliferation, enhanced ALP activity, and increased mRNA expression of BMP-2, osteocalcin, and RUNX2 (Fig. 6B and C). The number and area of calcium nodules were notably increased in BMSCs transfected with the miR-625-5p inhibitor (Fig. 6D). Additionally, we investigated the effect of miR-625-5p overexpression on quercetin-induced BMSC proliferation and osteogenic differentiation. miR-625-5p mimic was transfected into BMSCs to overexpress miR-625-5p and subsequently, BMSCs were treated with 10 μM quercetin; NC mimic was used as control. miR-625-5p levels were successfully increased by miR-625-5p mimic (Fig. 6A). The proliferation, ALP activity, and mRNA expression of BMP-2, osteocalcin, and RUNX2 were significantly decreased by miR-625-5p mimic (Fig. 6B and C). In addition, the number and area of calcium nodules were notably decreased by miR-625-5p mimic (Fig. 6D). Collectively, these results suggested that silencing miR-625-5p can promote BMSC proliferation and osteogenic differentiation, whereas miR-625-5p overexpression reverses these effect of quercetin.

### Quercetin promotes BMSC osteogenic differentiation by targeting the H19/ miR-625-5p axis and activating the Wnt/β-catenin signaling pathway

**Fig. 7:**
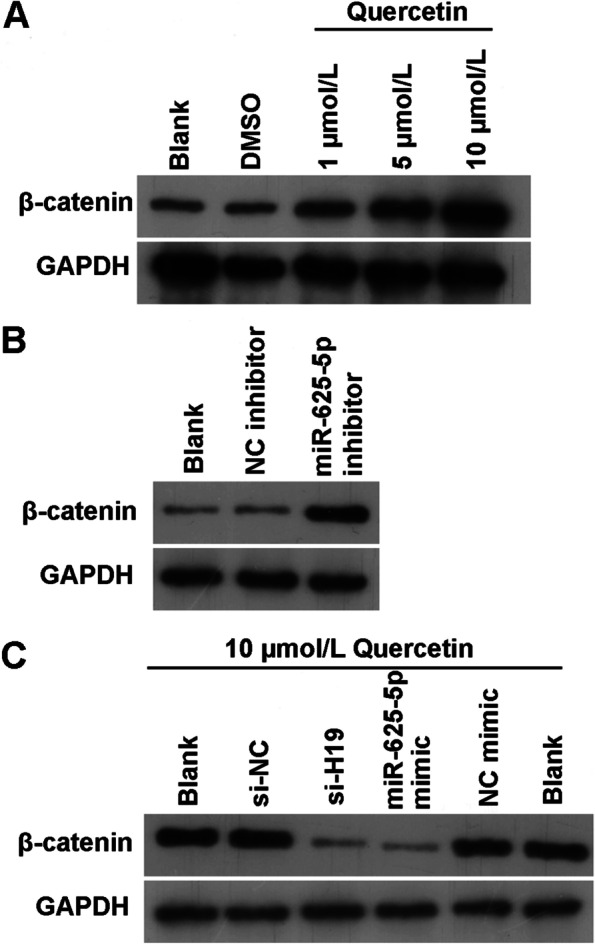
Wnt/β-catenin signaling pathway is involved in quercetin-induced BMSC osteogenic differentiation. **A** β-catenin protein levels increased in quercetin-induced BMSCs. **B** and **C** β-catenin protein levels in BMSCs were measured by western blotting (**B**) at 21 days after miR-625-5p inhibitor transfection, and **C** at 21 days after miR-625-5p mimic or si-H19 transfection

Finally, β-catenin protein levels were notably enhanced during quercetin-induced BMSC osteogenic differentiation (Fig. 7A). β-catenin protein level was significantly increased in response to treatment with miR-625-5p inhibitor (Fig. 7B). In addition, β-catenin protein levels decreased significantly after BMSCs were treated with si-H19 or miR-625-5p mimic followed by treatment with 10 μM quercetin (Fig. 7C).

## Discussion

Quercetin can heighten the BMSCs osteogenic differentiation and increase bone mineral density [6–9]. However, optimal concentration of quercetin treatment remains debatable. Treatment with 2, 2.5, and 5 μM quercetin promoted osteogenic differentiation of mouse BMSCs [7, 8]. Zhou et al reported that treatment with 2 μM quercetin promoted osteogenic differentiation of rat BMSCs, and this effect was even better than that observed following treatment with 5 or 10 μM quercetin [6]. Notoya et al reported that treatment with 5 and 10 μM concentration quercetin inhibited the osteogenic differentiation of Rob cells, whereas treatment with 0.1 and 1 μM concentration quercetin had no obvious effect on the proliferation and differentiation of Rob cells [22]. Treatment with 0.1, 1, and 10 μM concentration quercetin enhanced osteogenic differentiation of rat BMSCs, particularly at 10 μM [23]. We observed that quercetin treatment induced osteogenic differentiation in BMSCs and found that 10 μM quercetin was the optimal concentration to achieve these effects. The inconsistent effects of quercetin treatment on rat, mouse, and human BMSCs may be attributed to the differential tolerance of quercetin in different species. Casado-Díaz et al reported that BMSC differentiation medium supplemented with 10 μM quercetin decreased BMSC proliferation and differentiation, while BMSC differentiation medium supplemented with 0.1 μM quercetin promoted BMSC proliferation and differentiation [24]. Casado-Díaz’s study indirectly shows that quercetin promotes osteogenic differentiation. However, when present at a higher concentration (10 μM quercetin), in presence of another strong inducer of osteogenic differentiation (BMSC differentiation medium), it inhibits osteogenic differentiation. In our study, treatment with 1, 5, and 10 μM quercetin or BMSC differentiation medium treatment promoted BMSC proliferation and differentiation. Although based on our results we inferred that that 10 μM is the optimal quercetin concentration to induce osteogenic differentiation of BMSCs, the toxicity of high concentrations of quercetin (more than 2 μM) should be considered in animals and humans based on the above studies. Based on these observations, we propose that 1~2 μM quercetin may be the effective concentration to induce osteogenic differentiation without causing cytotoxicity. Further studies are warranted to validate these observations and inferences.

Previous studies also reported that H19 heightened osteogenic differentiation of BMSCs [14, 25, 26]. Consistently, H19 expression was upregulated during BMSC osteogenic differentiation in this study. Additionally, silencing H19 expression in BMSCs reversed the osteogenic differentiation-inducing effects of quercetin. This result suggested that H19 participates in the regulation of quercetin-induced BMSCs osteogenic differentiation. Functionally, H19 heightened BMSCs osteogenic differentiation by inhibiting the expression of miR-140-5p and miR-149 [14, 26]. In our study, H19 was found to interact with miR-625-5p during quercetin-induced BMSC osteogenic differentiation. The role of miR-625-5p on BMSC osteogenic differentiation was previously unclear. Our results revealed that silencing miR-625-5p promotes osteogenic differentiation of BMSCs, suggesting that miR-625-5p inhibits BMSCs osteogenic differentiation. Furthermore, miR-625-5p overexpression can reverse quercetin-induced osteogenic differentiation of BMSCs. Collectively, quercetin promotes BMSCs osteogenic differentiation by targeting the H19/miR-625-5p. In our study, H19 adsorbed miR-625-5p to regulate quercetin-induced osteogenic differentiation. Consistently with our results H19 can adsorb miRNAs include miR-140-5p, miR-149, and miR-532-3p to regulate osteogenic differentiation induced by other factors or drugs [14, 26, 27]. These results indicate that H19 regulated osteogenic differentiation can be further modulated by different factors by adsorbing different miRNAs.

H19 too can activate Wnt/β-catenin pathway to promote BMSCs osteogenic differentiation [28–30]. Quercetin can activate Wnt/β-catenin pathway to alleviate cerebral ischemia reperfusion injury and postmenopausal osteoporosis (PMOP) [9, 31]. Furthermore, quercetin treatment enhances the osteoblast differentiation of MC3T3-E1 cells via enhancing β-catenin protein level and activating the Wnt/β-catenin pathway [32]. These studies suggest that the Wnt/β-catenin pathway is the main downstream pathway in H19 and quercetin-induced osteogenic differentiation. We found that quercetin treatment or downregulation of miR-625-5p expression promoted β-catenin protein level in BMSCs, whereas upregulation of miR-625-5p expression or downregulation of H19 expression inhibited β-catenin protein level in quercetin-treated BMSCs. These results provide evidence that Wnt/β-catenin signaling is a downstream pathway regulated by quercetin and H19, consistent with previous studies [9, 28, 29, 31].

Clinically, osteogenic ability of BMSCs is weakened under some pathophysiological conditions, such as aging, menopause, trauma, and inflammation, all of which can lead to bone defects and osteoporosis [2–4, 33]. We found that quercetin treatment induced BMSC proliferation and osteogenic differentiation, suggesting that quercetin can be used for the clinical treatment of osteoporosis and bone defects. H19 expression is inhibited in patients with osteoporosis and bone defects, suggesting that H19 is a therapeutic target for these diseases [27, 34–36]. Since quercetin treatment increased H19 expression during quercetin-induced BMSCs osteogenic differentiation, quercetin may be used for the clinical treatment of osteoporosis and bone defects.

Although our study presents some credible data, it had some limitations. First, the target genes of miR-625-3p remain unclear. Additionally, quercetin may also activate the ERK and p38 MAPK pathways to enhance osteogenic differentiation of BMSCs, which can also be activated by H19 in cardiomyoblasts [23, 37], suggesting that the MAPK pathway may be another downstream pathway regulated by H19 in quercetin-induced osteogenic differentiation. Thus, to elucidate the relationship between quercetin, H19, and the ERK and p38 MAPK signaling pathways, further studies are warranted. Moreover, understanding the effect of quercetin on BMSC osteogenic differentiation in animal models and humans require further studies, and the nontoxic dose of quercetin remains to be confirmed.

## Conclusions

Our study demonstrated that H19 promoted, while miR-625-5p inhibited osteogenic differentiation of BMSCs. Quercetin promoted BMSC proliferation and osteogenic differentiation via the H19/miR-625-5p axis to activate the Wnt/β-catenin pathway. Additionally, 1~2 μM quercetin may be the effective concentration to induce osteogenic differentiation without causing cytotoxicity. However, the concentration and mechanism by which quercetin facilitates the osteogenic differentiation of BMSCs in vivo requires further exploration.

## Supplementary Information

**Additional file 1 : Figure 7.** Wnt/β-catenin signaling pathway participated in quercetin-induced BMSCs osteogenic differentiation. (A) β-catenin protein level in quercetin-treated BMSCs was enhanced after treatment at 21 days. (B) β-catenin protein level was promoted by miR-625-5p inhibitor transfection at 21 days. The experiment was repeated twice (repetition 1 include the hole 1, 2, and 3; repetition 2 include the hole 4, 5, and 6). And we chose the result of experiment repetition 2 as the representative image in manuscript. (C) β-catenin protein level was reduced in quercetin-induced BMSCs after miR-625-5p mimic or si-H19 transfection at 21 days. β-catenin protein was measured by western blot

## Data Availability

The datasets used and/or analyzed in this study are available from the corresponding author upon reasonable request.

## Abbreviations

ALP: Alkaline phosphatase
AMP: Adenosine 5'-monophosphate
BMP-2: Bone morphogenetic protein 2
BMSCs: Bone marrow mesenchymal stem cells
lncRNA: Long noncoding RNA
RUNX2: Runt-related transcription factor 2
