# Supplementary material for: Quercetin promotes bone marrow mesenchymal stem cell proliferation and osteogenic differentiation through the H19/miR-625-5p axis to activate the Wnt/β-catenin pathway
Source: BMC Complement Med Ther. 2021 Sep 30;21:243. doi: 10.1186/s12906-021-03418-8 (PMC8485455; doi:10.1186/s12906-021-03418-8)
Supplement: Supplementary file 1 — Additional file 1 : Figure 7. Wnt/β-catenin signaling pathway participated in quercetin-induced BMSCs osteogenic differentiation. (A) β-catenin protein level in quercetin-treated BMSCs was enhanced after treatment at 21 days. (B) β-catenin protein level was promoted by miR-625-5p inhibitor transfection at 21 days. The experiment was repeated twice (repetition 1 include the hole 1, 2, and 3; repetition 2 include the hole 4, 5, and 6). And we chose the result of experiment repetition 2 as the representative image in manuscript. (C) β-catenin protein level was reduced in quercetin-induced BMSCs after miR-625-5p mimic or si-H19 transfection at 21 days. β-catenin protein was measured by western blot [file 12906_2021_3418_MOESM1_ESM.pdf]

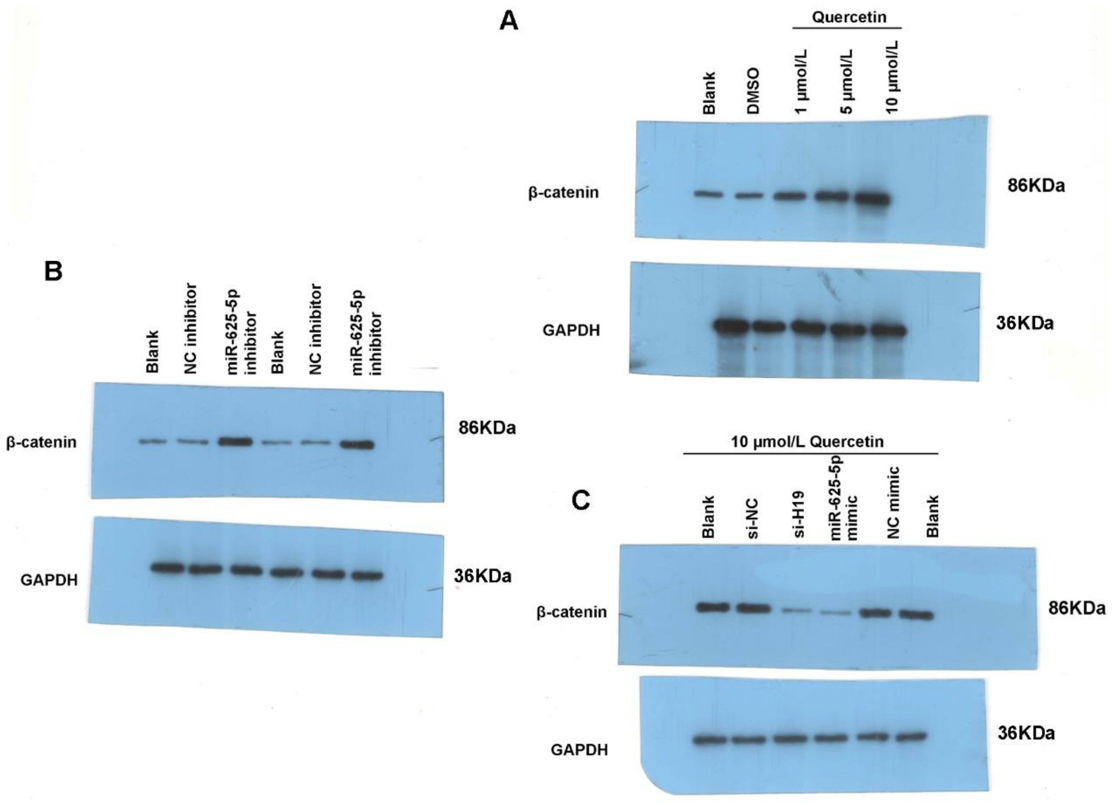

Figure 7 Wnt/β-catenin signaling pathway participated in quercetin-induced BMSCs osteogenic differentiation. (A) β-catenin protein level in quercetin-treated BMSCs was enhanced after treatment at 21 days. (B) β-catenin protein level was promoted by miR-625-5p inhibitor transfection at 21 days. The experiment was repeated twice (repetition 1 include the hole 1, 2, and 3; repetition 2 include the hole 4, 5, and 6). And we chose the result of experiment repetition 2 as the representative image in manuscript. (C) β-catenin protein level was reduced in quercetin-induced BMSCs after miR-625-5p mimic or si-H19 transfection at 21 days. β-catenin protein was measured by western blot
